# G Protein-Coupled Receptor Kinase 2 (GRK2) as a Potential Therapeutic Target in Cardiovascular and Metabolic Diseases

**DOI:** 10.3389/fphar.2019.00112

**Published:** 2019-02-19

**Authors:** Cristina Murga, Alba C. Arcones, Marta Cruces-Sande, Ana M. Briones, Mercedes Salaices, Federico Mayor Jr.

**Affiliations:** ^1^ Departamento de Biología Molecular, Centro de Biología Molecular Severo Ochoa (UAM-CSIC), Universidad Autónoma de Madrid, Madrid, Spain; ^2^ CIBER de Enfermedades Cardiovasculares (CIBERCV), Instituto de Salud Carlos III, Madrid, Spain; ^3^ Instituto de Investigación Sanitaria La Princesa, Madrid, Spain; ^4^ Departamento de Farmacología, Universidad Autónoma de Madrid (UAM), Madrid, Spain; ^5^ Instituto de Investigación Hospital Universitario La Paz (IdiPAZ), Madrid, Spain

**Keywords:** GRK2, GPCR, cardiovascular, obesity, NAFLD, insulin resistance, inhibitors

## Abstract

G protein-coupled receptor kinase 2 (GRK2) is a central signaling node involved in the modulation of many G protein-coupled receptors (GPCRs) and also displaying regulatory functions in other cell signaling routes. GRK2 levels and activity have been reported to be enhanced in patients or in preclinical models of several relevant pathological situations, such as heart failure, cardiac hypertrophy, hypertension, obesity and insulin resistance conditions, or non-alcoholic fatty liver disease (NAFLD), and to contribute to disease progression by a variety of mechanisms related to its multifunctional roles. Therefore, targeting GRK2 by different strategies emerges as a potentially relevant approach to treat cardiovascular disease, obesity, type 2 diabetes, or NAFLD, pathological conditions which are frequently interconnected and present as co-morbidities.

## Introduction

G protein-coupled receptor kinases (GRKs) were originally identified as key regulators of G protein-coupled receptor (GPCR) function. GPCRs are activated by a wide variety of stimuli and comprise the largest family of membrane receptors. GPCRs are crucially involved in a multitude of physiological process, and their dysregulation contributes to many diseases, having thus become the targets of ~35% of prescription drugs (Sriram and Insel, 2018). A fundamental characteristic of GPCR biology is that agonist-stimulated receptors, in addition to trigger activation of heterotrimeric G proteins and their classical downstream effectors, are specifically phosphorylated by GRKs, which in turn promotes binding of β-arrestin proteins to GPCRs thus inhibiting further interactions with G proteins, a process termed desensitization. Moreover, the fact that β-arrestins act as scaffold for proteins of the endocytic machinery and for many other signal transduction partners leads to transient receptor internalization and to a second wave of G protein-independent GPCR signaling cascades based on β-arrestin-orchestrated signalosomes. Therefore, GRK dosage and activity is a key feature that determines the extent of GPCR desensitization and internalization and the balance between G protein- and β-arrestin-dependent branches of GPCR signaling [reviewed in (Ranjan et al., 2016; Smith and Rajagopal, 2016; Chen et al., 2018)].

The mammalian GRK family entails seven members classified into three subfamilies: visual GRKs (GRK1, also known as rhodopsin kinase, and GRK7), the GRK2 subfamily (GRK2 and GRK3), and the GRK4 subfamily (GRK4, GRK5 and GRK6). GRK2, 3, 5, and 6 are ubiquitously expressed at varying levels, whereas GRK4 displays a more restricted tissue expression pattern and GRK1 and 7 are primarily present in specific retinal cells (Ribas et al., 2007; Mushegian et al., 2012; Watari et al., 2014). Importantly, accumulating evidence indicates that GRKs can act as signal transducers by themselves, displaying a repertoire of substrates and/or interacting partners beyond GPCR and also a more general regulatory role in a variety of signaling processes (Penela et al., 2010b; Mushegian et al., 2012; Watari et al., 2014; Nogues et al., 2017).

This multifunctionality is particularly evident for GRK2, which shows a complex array of non-GPCR substrates and cellular interactors, including receptor tyrosine kinases and their downstream molecules (EGFR, PDGFR, IRS1), signal transduction kinases (p38Mapk, AMPK, PI3K/Akt, MEK1), G protein subunits and modulators (Gαq, Gβγ, phosducin, RhoaA, RalA, EPAC1), transcription factors and their regulatory proteins (Smad2/3, IκBα), cytoskeletal proteins and modulators (ezrin, tubulin, GIT1, HDAC6), ubiquitin ligases (Mdm2, Nedd4–2), or different enzymes relevant for signaling (Pin1, eNOS) [reviewed in (Penela et al., 2010b; Evron et al., 2012; Mushegian et al., 2012; Nogues et al., 2017; Mayor et al., 2018)]. Moreover, altered GRK2 levels and functionality have been found in patient samples and/or in animal models of relevant pathological situations such as heart failure, hypertension, obesity and insulin resistance-related situations, non-alcoholic fatty liver disease (NAFLD), pain, inflammation, and some types of cancer, where anomalous GRK2 dosage or activity appears to contribute to disease progression *via* cell type and context-specific molecular mechanisms (Mushegian et al., 2012; Penela et al., 2014; Gurevich et al., 2016; Hullmann et al., 2016; Cannavo and Koch, 2018; Cruces-Sande et al., 2018; Mayor et al., 2018; Nogues et al., 2018; Wang et al., 2018). Interestingly, several cardiovascular diseases as well as obesity and type 2 diabetes-related disorders, clinical conditions often interrelated as co-morbidities, converge in displaying increased GRK2 levels, pointing at the inhibition of GRK2 as an attractive therapeutic target. We summarize in this review the physiopathological roles of GRK2 in cardiovascular and metabolic diseases and focus on potential strategies to downregulate GRK2 functions based on our current knowledge about the structural features and mechanisms of regulation of this protein.

## Molecular Mechanisms Controlling GRK2 Activation and Functionality

**Figure 1:**
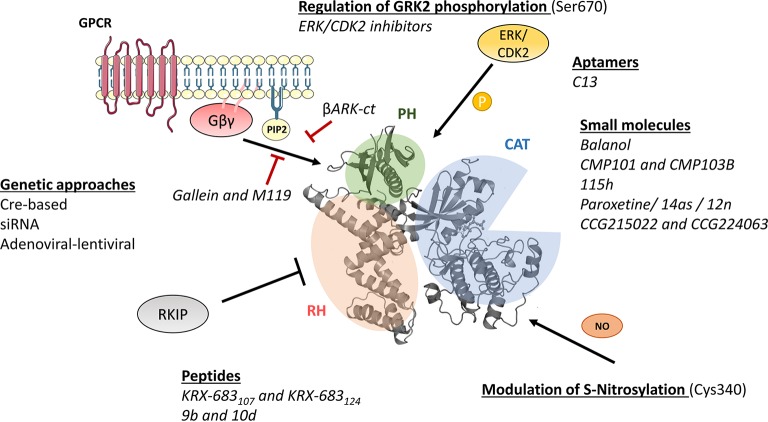
Molecular mechanisms of GRK2 activation and functionality relevant for the design of therapeutic strategies. GRK2 dosage has been altered in different preclinical models by using global or tissue-specific Cre-based depletion methodologies, siRNA technology, and also adenoviral and lentiviral transfer of GRK2-specific silencing constructs. In addition to small molecule and aptamer compounds that able to keep the kinase in inactive conformations, other strategies to block GRK2 activation are based on the use of peptide sequences, fragments of its domains (βARKct), or small molecules (gallein, M119) in order to interfere with known GRK2 activators as GPCR and Gβγ subunits. Other strategies may be based on the interaction of GRK2 with inhibitory proteins such as RKIP, S-nitrosylation of specific residues in the catalytic domain, or *via* modulation of GRK2 phosphorylation at residues relevant for determining the substrate repertoire of GRK2. See text for details.

As the rest of the GRK isoforms, GRK2 is a multidomain protein organized in several domains and regions. The elucidation of the structure of GRK2 alone (Lodowski et al., 2005) in complex with Gβγ subunits (Lodowski et al., 2003) or with both Gβγ and Gαq subunits (Tesmer et al., 2005) and the comparison with the available structures of other GRKs (Komolov and Benovic, 2018) has provided key insights into GRK2 activation mechanisms. All GRKs are serine/threonine kinases that belong to the large AGC kinase family and share a catalytic domain displaying the characteristic bilobular fold of protein kinases, with high similarity to other AGC members, such as PKA, PKB, and PKC (Pearce et al., 2010). This catalytic core is preceded by a domain displaying homology to RGS proteins (thus termed RH domain) that, in the case of GRK2 subfamily members, has been shown to specifically interact with Gαq/11 subunits, thus blocking its interaction with their effectors (Carman et al., 1999; Sanchez-Fernandez et al., 2016). The RH domain displays at its far N-terminus a N-terminal helix (αN) characteristic of GRKs and important for receptor recognition. The C-terminal region is more variable among GRKs, but in all cases it is key for the localization to the plasma membrane. The C-terminal region of GRK2 and GRK3 contains a pleckstrin homology domain (PH) that able to interact with membrane lipids such as the phospholipid PIP2 and also with free Gβγ subunits (Homan and Tesmer, 2014; Nogues et al., 2017) (Figure 1).

Importantly, GRKs show mechanisms of activation that are different to those of AGC kinases. In most AGC kinases, transitions from inactive to active conformations imply phosphorylation of regulatory motifs at the activation segment/loop located in the large kinase lobe and at the hydrophobic motif found C-terminal to the small kinase lobe. Phosphorylation of these sites directs the closure of catalytic lobes and stabilizes the active conformation of the critical αC helix (Pearce et al., 2010). However, such phosphorylated regulatory motifs are absent in GRK2, and this protein thus requires conformation-induced rearrangements to become active. GRK2 activation is based on the dynamic interactions of its αN-helix and the RH and PH domains among themselves and with activating partners such as agonist-occupied GPCR, Gβγ subunits, and PIP2, eventually leading to allosteric rearrangement of the functionally relevant AST loop and kinase domain closure (Homan and Tesmer, 2014; Nogues et al., 2017; Komolov and Benovic, 2018).

The recent co-crystallization of GRK5 with the β2AR (Komolov et al., 2017) indicates that GRKs would display high structural plasticity, with large conformational changes in the GRK5 RH/catalytic domain interface upon GPCR binding. In this model, the RH domain would serve as a docking site for GPCRs and help kinase activation *via* transient contacts of the RH bundle and kinase subdomains (Komolov and Benovic, 2018). Other studies support an important role for the RH domain of GRKs in GPCR interaction (Dhami et al., 2004; Baameur et al., 2010, 2014; He et al., 2017). In addition to the RH domain, the αN-helix is also relevant to stabilize the kinase domain closure in a process regulated by GPCR binding (Singh et al., 2008; Pao et al., 2009; Boguth et al., 2010; Beautrait et al., 2014). Additionally, other motifs located within the catalytic domain of GRK2 might also participate in GPCR binding (Gan et al., 2000, 2004).

The identification of residues and regions critical for receptor docking and/or allosteric kinase activation may help understand the preferential modulation of GPCR by certain GRKs and also guide the design of inhibitory strategies. On the other hand, although the current model explains how closure of the kinase domain and subsequent GRK2 activation is achieved in a GPCR and Gβγ/phospholipid-dependent manner, it is important to ascertain how GRK2 might be activated to trigger phosphorylation of the increasing and heterogeneous repertoire of non-GPCR and non-membrane GRK2 substrates.

In this regard, it has been shown that GRK2 functionality can be influenced by post-translational modifications in both the RH domain and the C-terminal region of the kinase (Penela et al., 2010a, 2014; Lafarga et al., 2012; Nogues et al., 2017). GRK2 can be phosphorylated by c-Src or EGFR on tyrosine residues located in the αN-helix (Tyr13) and within the RH region (Tyr-86 and Tyr-92), leading to enhanced catalytic activity toward both membrane receptors and soluble substrates (Sarnago et al., 1999; Penela et al., 2003; Chen et al., 2008; Sun et al., 2017), suggesting an allosteric effect on the kinase domain *via* contacts of these residues with the AST region in the kinase lobes (Beautrait et al., 2014). On the other hand, second messenger-governed kinases, such as PKA and PKC, respectively, phosphorylate GRK2 at Ser685 enhancing its ability to bind to Gβγ and the activated GPCR (Cong et al., 2001) or on Ser29 to increase GRK2 activity toward GPCR but not soluble peptides (Krasel et al., 2001).

Phosphorylation of GRK2 at Ser670 by different kinases such as ERK1/2 (Elorza et al., 2003) or CDK2 (Penela et al., 2010c) appears to play a very relevant modulatory role. This residue lies at the far C-terminus of GRK2 within the Gβγ-binding domain, and its phosphorylation impairs the interaction with Gβγ subunits, thus hindering GRK2 translocation to the plasma membrane and activity toward GPCR (Pitcher et al., 1999; Elorza et al., 2000). Strikingly, GRK2 Ser670 phosphorylation can promote changes in substrate specificity, association with partners and subcellular localization. Stimuli or context-specific ERK1/2-dependent GRK2 Ser670 phosphorylation is required to enable GRK2 to phosphorylate HDAC6 (Lafarga et al., 2012; Nogues et al., 2016), disrupts GRK2 interaction with GIT1 (Penela et al., 2008), or directs localization to the mitochondrial outer membrane *via* enhanced interaction with the chaperone Hsp90 (Chen et al., 2013). Thus, S670 phosphorylation plays a central role in the modulation of GRK2 functional features.

As for other mechanisms that able to control GRK2 activity, GRK2 can undergo S-nitrosylation of the Cys residue 340 within its core catalytic domain, obstructing kinase activity toward GPCRs (Whalen et al., 2007). On the other hand, GRK2 binds to clathrin, caveolin, or RKIP, which appear to participate in keeping GRK2 activity at bay at specific cellular locations (Gurevich et al., 2012; Schmid et al., 2015).

Finally, GRK2 expression levels are tightly regulated by a variety of mechanisms. GRK2 is rapidly degraded by the proteasome pathway in both basal and GPCR-stimulated conditions (Penela et al., 1998; Penela et al., 2003). GRK2 ubiquitination and turnover is enhanced by GPCR activation through complex mechanisms encompassing combined phosphorylation of GRK2 by c-Src and MAPK in a β-arrestin-dependent manner (Penela et al., 1998, 2001; Elorza et al., 2003), being Mdm2 a key E3 ligase involved in GRK2 proteolysis by the proteasome (Salcedo et al., 2006). While regulation of stability seems to be the main pathway controlling GRK2 dosage, changes in mRNA transcription have also been reported in several pathological conditions, including hypertension, cardiac hypertrophy, and heart failure (Cannavo and Koch, 2018; Mayor et al., 2018). However, relatively little is known about the mechanisms governing promoter activity and transcript stability of GRK2 or regarding the participation of miRNAs in GRK2 regulation, although miR-K3, a Kaposi’s sarcoma-associated herpesvirus (KSHV) miRNA, has been shown to repress GRK2 expression (Hu et al., 2015). A better knowledge of the mechanisms of modulation of GRK2 protein turnover and expression would help to design strategies to control GRK2 dosage in pathological settings.

## Physiopathological Roles of GRK2

**Figure 2:**
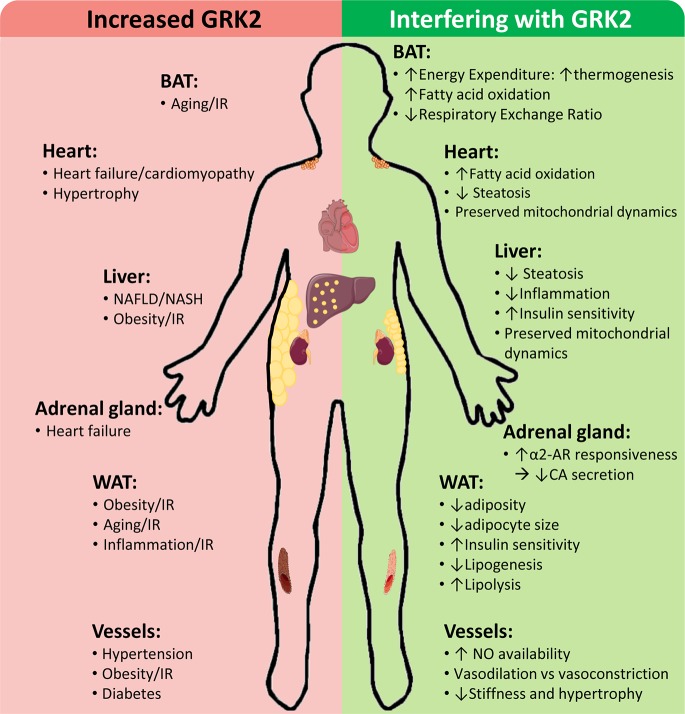
Cardiovascular and metabolic disease-related contexts with increased GRK2 levels and effects of interfering GRK2 expression or functionality. Enhanced GRK2 levels have been reported in different tissues and cell types of patients and/or preclinical models of the indicated situations associated with cardiovascular and metabolic diseases. On the other hand, interfering GRK2 functionality in these settings in preclinical models can modulate different relevant cellular processes implicated in disease development and progression. See text for details.

GRK2 levels and activity are reportedly increased in different tissues of patients and/or in preclinical models in cardiovascular and metabolic disease-related contexts, contributing to disease progression by a variety of mechanisms, whereas GRK2 inhibition plays a protective role (Figure 2).

### GRK2 in Cardiac Function and Pathology

GRK2 has a relevant role in the control of cardiac function and enhanced GRK2 expression has been reported in the failing human hearts and in experimental models of heart failure (HF) in contexts of both chronic hypertensive and ischemic disease. Accordingly, genetic GRK2 deletion or pharmacological inhibition is cardioprotective in animal models recapitulating these pathological settings [reviewed in (Penela et al., 2006; Schumacher et al., 2015; de Lucia et al., 2018)].

Cardiac GRK2 mRNA and/or protein levels appear to be increased in HF patients with dilated or ischemic cardiomyopathy as a consequence of sympathetic nervous system hyperactivity in such situations. Augmented release of catecholamines is an early compensatory mechanism triggered in response to myocardial damage and dysfunction in order to maintain cardiac output *via* β-adrenergic-mediated effects in cardiac contractility. Enhanced GRK2 levels in the heart in such situations would initially help compensate such β-adrenergic overdrive. However, continued adrenergic stimulation and cardiac GRK2 overexpression are maladaptive, finally leading to decreased contractility and altered cardiac cell metabolism and survival (Ungerer et al., 1993; Sato et al., 2015b; de Lucia et al., 2018). Of note, in such contexts, the increased catecholamine release in adrenal glands triggers a “damaging cycle” by fostering GRK2 upregulation also in the chromaffin adrenal cells, which prevents α_2_-adrenergic receptor-mediated feedback inhibition of adrenal catecholamine secretion thus perpetuating the neurohormonal overdrive and boosting GRK2- and β-adrenergic-mediated maladaptive actions (Lymperopoulos et al., 2007, 2016; Lymperopoulos, 2011).

Although such long-term maladaptive increase in GRK2 expression is well established in different experimental models, a decrease in GRK2 levels *via* proteasomal degradation has been reported in the subepicardial border and the infarct zone at 6 and 24 h after ligation of the coronary artery in dogs, and treatment with proteasome inhibitors shown to prevent GRK2 degradation and result in significant cardioprotection against malignant ventricular tachyarrhythmias (Yu et al., 2000, 2005; Huang et al., 2008). Therefore, a better knowledge of the time course of changes in cardiac GRK2 levels in pathological situations and its functional impact is required to better define the correct timing of therapeutic strategies targeting GRK2.

The functional role of GRK2 in cardiac tissue involves both modulation of GPCR and interactions with other signaling molecules. GRK2 overexpression in transgenic mice attenuates βAR signaling, whereas hemizygous GRK2 mice show an enhanced response to adrenergic agonists (Koch et al., 1995; Rockman et al., 1998). On the other hand, transverse aortic constriction and continued angiotensin II-mediated stimulation of Gq-coupled AT1 receptors cause increased GRK2 expression in the context of cardiac hypertrophy, and GRK2 has also been shown to modulate angiotensin II-mediated contraction by directly interacting with Gαq (Schumacher et al., 2016). GRK2 may also regulate the functionality of other relevant cardiac GPCRs such as GLP-1, adiponectin, k-opioid, or purinergic receptors (Heusch, 2015; Wang et al., 2015; Chen et al., 2017; Mayor et al., 2018).

In addition, GRK2 has emerged as an important modulator of cardiac insulin signaling, which is key for a balanced glucose and fatty acid metabolism in the heart and has cardioprotective features (Lucas et al., 2015; Hullmann et al., 2016; Riehle and Abel, 2016). Increased GRK2 levels worsen glucose uptake after ischemic injury *via* IRS1 phosphorylation, whereas cardiac-specific GRK2 knockout mice show enhanced heart glucose metabolism (Ciccarelli et al., 2011). Notably, a high fat diet (HFD) promotes cardiac GRK2 accumulation leading to impaired cardiac insulin sensitivity and reduced stimulation of the cardioprotective PI3K/Akt pathway, whereas this effect was prevented in 9-month-old hemizygous GRK2+/− mice, which showed a preserved insulin-dependent downstream signaling and an overall cardioprotective gene expression reprogramming (Lucas et al., 2014, 2015). Therefore, situations of neurohumoral activation and of obesity/insulin-resistance converge in promoting cardiac GRK2 upregulation, which emerges as a potential factor linking insulin-resistant pathological conditions and heart failure. In this regard, in a long-term obesity-induced cardiac remodeling model, reduced GRK2 dosage in hemizygous GRK2+/− mice preserves the activation of the PKA/CREB and AMPK pathways downstream of different cardiac GPCR, maintains the expression of key cardioprotective metabolic enzymes and mitochondrial proteins, and thus safeguards these animals from obesity-induced cardiomyocyte hypertrophy, fibrosis, and steatosis (Lucas et al., 2016). Also supporting a key role for GRK2 in insulin/GPCR pathways crosstalk, hyperinsulinemia associated to type 2-diabetes fosters β2AR phosphorylation leading to decreased βAR-regulated cardiac contractility, in a manner dependent on IRS1/IRS2, PKA, and GRK2 activity (Fu et al., 2014), this effect being potentiated upon GRK2 over expression and prevented upon GRK2 inhibition (Wang et al., 2017a).

These data point to a scenario in which GRK2 levels act as an integrating hub controlling both myocardial contractile function and cardiac metabolism. In fact, GRK2 levels appear to control such key aspects of cardiac function by molecular mechanisms additional to those already described. GRK2 localizes to cardiac mitochondrial fractions upon ERK1/2 phosphorylation in ischemic contexts, which favors GRK2/Hsp90 interaction and mitochondrial targeting. Upregulated mitochondrial GRK2 seems to be required for mitochondrial-dependent death pathway signaling and facilitates calcium-induced opening of the mitochondrial permeability transition pore (Chen et al., 2013). Moreover, the mitochondrial GRK2 pool appears to enhance superoxide levels and to diminish substrate utilization for energy production (Sato et al., 2015a), whereas enhanced kinase levels in cardiomyocytes potentiate oxidative stress and apoptosis (Theccanat et al., 2016) and contribute to impaired fatty acid uptake and oxidation (Pfleger et al., 2018). However, in other cell models, a positive role for GRK2 in mitochondrial biogenesis and ATP generation (Fusco et al., 2012) and a potential protective effect of GRK2 shuttling to the mitochondria by modulating fusion and recovery of this organelle in response to acute cell damage have been suggested (Sorriento et al., 2014; Franco et al., 2018). Therefore, more research is needed to define the role of mitochondrial GRK2 in both homeostasis and specific pathological contexts. Additionally, in transgenic models displaying increased cardiac GRK2, an impairment of the cardioprotective eNOS pathway and reduced NO bioavailability is observed in cardiac cells, thus promoting increased myocardial injury in ischemia/reperfusion mice models by mechanisms involving mutual inhibition of GRK2 and eNOS (Huang et al., 2013).

It is also worth noting that altered GRK2 levels in other cardiac cell types may contribute to heart dysfunction. An increase in GRK2 expression predominantly in endothelial cells was reported in the heart of rats 7 days after induction of myocardial infarction (Vinge et al., 2001). In addition, increased GRK2 levels in cardiac fibroblasts contribute to enhanced collagen synthesis and fibrosis and appear to favor maladaptive remodeling upon ischemia/reperfusion injury (Woodall et al., 2016), whereas inhibition of the Gβγ-GRK2 axis limits pathological myofibroblast activation after myocardial ischemia (Travers et al., 2017).

On the other hand, augmented GRK2 levels have been reported in peripheral blood samples in HF (Rengo et al., 2016) or acute myocardial infarction (Santulli et al., 2011) patients, but the potential pathophysiological impact of such altered dosage remains to be determined. Early and balanced recruitment of cells of the innate immune system (monocytes/macrophages, mast cells, and neutrophils) is key for initiating cardiac remodeling and repair after heart injury (Frangogiannis, 2014). Given the reported ability of GRK2 to regulate inflammatory responses and NFκB signaling (Sorriento et al., 2015; Steury et al., 2017) as well as chemokine and other GPCR involved in leukocyte trafficking (Grisanti et al., 2016), it is tempting to suggest that altered GRK2 levels in circulating cells may modulate the timing or intensity of the immune response triggered upon cardiac injury.

In sum, enhanced cardiac GRK2 dosage in pathological conditions is expected to simultaneously alter key pathways controlling cardiac physiopathology, including βAR and GPCR signaling, mitochondrial function, cardiac insulin signaling, and also metabolic and survival cascades or NO bioavailability, ultimately contributing to altered contractility, metabolic and energetic derangement, pathological gene expression reprogramming, maladaptive myocardial remodeling, and progression to heart failure.

These data put forward cardiac GRK2 inhibition as an attractive therapeutic strategy. In fact, cardiomyocyte-specific knockout of GRK2 in mice protects (Fan et al., 2013) injury promoted by ischemia/reperfusion while damage is increased in cardiac transgenic mice overexpressing the kinase (Brinks et al., 2010), by mechanisms reportedly related to proapoptotic signaling linked to increased GRK2 mitochondrial pools. Moreover, GRK2 deletion 10 days after myocardial infarction (Raake et al., 2008) or treatment with the GRK2 inhibitor paroxetine (see below) started 2 weeks after injury (Schumacher et al., 2015) improves cardiac function and reduces adverse ischemic remodeling in mice. On the other hand, adenoviral-mediated delivery of the C-terminal region of GRK2 (βARKct) also has a protective effect in heart failure and acute myocardial infarction settings in murine and pig models (reviewed in Sato et al., 2015b; Cannavo and Koch, 2018; de Lucia et al., 2018) by mechanisms likely involving prevention of the activation of GRK2 by Gβγ subunits (Rudomanova and Blaxall, 2017b).

A better knowledge of the interactome of GRK2 and its modulation in the different cardiac cell types, of the molecular mechanisms and stimuli leading to increased GRK2 expression in settings of cardiac hypertrophy, ischemia/reperfusion, and post-infarction remodeling, and of the detailed temporal pattern of such changes would help to fine-tune the therapeutic strategies targeting this protein.

### Vasculature

Vascular tone is regulated by vasodilator and vasoconstrictor factors mainly released by endothelial cells (EC) in response to mechanical or chemical stimuli. The imbalance between these substances leads to the endothelial dysfunction and/or altered vasoconstrictor responses observed in cardiovascular diseases. Among the different systems involved in vascular dysfunction are effectors of the sympathetic nervous and renin angiotensin systems, endothelin-1, and prostanoids that can signal through specific GPCR leading to reduced NO bioavailability and endothelial dysfunction by different mechanisms (Vanhoutte, 2018). These include, among others, modulation of the expression/activity of the endothelial NO synthase (eNOS), eNOS uncoupling due to substrate or cofactor deficiency, and alterations in eNOS activation through its phosphorylation by different kinases including the PI3K/Akt pathway (Vanhoutte et al., 2016; Vanhoutte, 2018). In addition, activation of many GPCRs controls vascular smooth muscle cells (VSMC) proliferation and migration as well as extracellular matrix deposition (Althoff and Offermanns, 2015).

Changes in GRK2 levels and/or activity can mediate important effects in vascular function and structure that have been classically explained by GRK2-dependent desensitization of different GPCRs (Brinks and Eckhart, 2010) such as angiotensin II (AngII), endothelin, or adrenergic receptors. Overall, different studies describe that a partial deficiency or inhibition of GRK2 differentially alters vasoconstrictor responses to GPCR agonists (Rainbow et al., 2018), while vasodilator effects seem to be more generally increased. This apparently preferential desensitization by GRK2 of vasodilation versus vasoconstriction signaling has been invoked to explain the effect of upregulated GRK2 levels seen in human and murine hypertension (Eckhart et al., 2002; Izzo et al., 2008; Cohn et al., 2009; Santulli et al., 2013). In this line, studies in sinusoidal endothelial cells from injured livers demonstrated that Akt physically interacts with GRK2, which inhibits Akt activation and NO production (Liu et al., 2005). Thus, the increased expression of GRK2 observed in vessels from different mouse models of vascular or metabolic diseases (Taguchi et al., 2011a, 2012b) can be an underlying factor that decreases NO bioavailability and contributes to endothelial dysfunction (Lucas et al., 2015). For instance, chronic AngII treatment of C57Bl6 mice upregulates GRK2 in vessels, leads to inhibition of the Akt/eNOS pathway, and thus reduces NO production both basally and after AngII-infusion that finally results in endothelial dysfunction (Avendano et al., 2014). Interestingly, some of these differences appear to be gender-dependent (Taguchi et al., 2012a). More importantly, GRK2 inhibition or partial GRK2 deletion improved the endothelial dysfunction observed in obese/diabetic (Taguchi et al., 2011b, 2012c, 2013) or hypertensive (Avendano et al., 2014) animal models by restoring the impaired Akt/eNOS pathway and NO availability in a process in which glucose homeostasis may be implicated (Taguchi et al., 2017). Overall, these studies consolidate GRK2 as a genuine negative modulator of NO bioavailability. However, as mentioned above, recent evidence suggest that a reciprocal negative regulation exists between GRK2 and NO with increased NO bioavailability being an endogenous inhibitor of this kinase by S-nitrosylation of Cys340 of GRK2 [reviewed in (Cannavo and Koch, 2018)] pointing to a feedforward relationship between NO and GRK2. Interestingly, some recent reports have described that a more profound and global GRK2 knockdown may be detrimental due to enhanced renin- and AT1R-mediated reactive oxygen species (ROS) production that can cause renal damage (Tutunea-Fatan et al., 2018) and the development of hypertension with age (Tutunea-Fatan et al., 2015). Together, these findings suggest that partial or local rather than generalized GRK2 targeting might be considered when increased NO levels and a correction of endothelial dysfunction need to be achieved.

Vessels from patients or animal models of obesity and metabolic syndrome frequently display hypertrophic remodeling with particular differences depending on the vascular bed (Briones et al., 2014). This vascular remodeling seems to be influenced by hemodynamic factors such as hypertension and most often can be reversed at least in part by pharmacological blockade of the renin-angiotensin-aldosterone or endothelin systems (Briones et al., 2014). The role of GRK2 in the structural and mechanical alterations in vessels is only beginning to be elucidated and might depend on the cell-specific location of GRK2 or on the experimental model. Thus, a partial overall GRK2 deletion prevents vascular hypertrophy and increased vessel stiffness observed in the AngII-induced hypertension model (Avendano et al., 2014). However, endothelial-specific depletion of GRK2 in Tie2-CRE/GRK2(fl/fl) mice leads to structural abnormalities that were reverted by a ROS scavenger (Ciccarelli et al., 2013), and an impaired angiogenic response with immature vessels was observed in these animals and in GRK2+/− mice (Rivas et al., 2013). The mechanisms responsible for these GRK2-mediated effects in vascular structure and development are not fully understood, but GRK2 and β-arrestins participate in the modulation of arterial smooth muscle purinergic signaling (Morris et al., 2011) and of agonist-stimulated VSMC migration through activation of proliferative and promigratory MAPK such as ERK1/2 (Morris et al., 2012). Conversely, a negative role for GRK2 in VSMC proliferation has also been shown (Peppel et al., 2000; Guo et al., 2009). In sum, the results presented in this section highlight a potentially novel strategy for the regulation of vascular dysfunction through GRK2 targeting.

### Adipose Tissues

As a combination of different repositories, the adipose tissue can be considered a multi-depot organ located mainly in two compartments: subcutaneous and visceral (Cinti, 2005). Adipose depots can also be differentiated into white and brown adipose tissue according to their specific physiological roles and morphological appearance.

### White Adipose Tissue

Classically considered as a mere energy store, the white adipose tissue (WAT) forms a large organ devoid of a specific shape and size but with a large physiological plasticity (Cinti, 2012) and important endocrine and homeostatic functions (Trayhurn and Beattie, 2001). Structural and/or functional variations in WAT together with changes of its fat load are key features correlating with metabolic alterations (Primeau et al., 2011; McLaughlin, 2012). In this regard, insulin is a critical regulator of the most important aspects of adipocyte biology. This hormone promotes triglyceride storage within WAT by promoting adipocyte differentiation and by stimulating glucose uptake and triglyceride synthesis while inhibiting lipolysis Thus, maintaining the integrity of insulin signaling is crucial for an adequate physiological role of adipocytes.

In this line, GRK2 has been unveiled as an important regulator of insulin signaling in different insulin-target tissues including WAT. In fact, GRK2 hampers insulin-mediated glucose uptake in 3T3L1 adipocytes by several mechanisms including interfering with Gαq/11 (Usui et al., 2005) and sequestering IRS1 in a process that is independent of its kinase activity (Garcia-Guerra et al., 2010). On the other hand, GRK2 can also act as a modulator of overall adiposity and fat mass accretion [reviewed in (Mayor et al., 2011)]. Aged or HFD-fed GRK2+/− mice show a decreased size of white adipocytes (Garcia-Guerra et al., 2010) and a reduced expression of enzymes involved in lipogenesis (Vila-Bedmar et al., 2012). Moreover, tamoxifen-induced GRK2 depletion during a HFD reduces adipose WAT mass and adipocyte size and increases markers of lipolysis as well as the *ex vivo* and *in vivo* lipolytic response of this tissue even after overweight and IR have already been established (Vila-Bedmar et al., 2015). Furthermore, insulin resistance, glucose levels, and GRK2 expression emerge as strongly associated variables in a homeostasis model assessment of adipose-derived stem cells obtained from lean and obese human patients (Ceperuelo-Mallafre et al., 2014). These data suggest that GRK2 can play a relevant role in the establishment of insulin-resistant states in murine and human WAT and that these states can be overturned by genetic ablation of GRK2, thus putting forward that interfering with GRK2 could be a good strategy to revert the damaging effects of a dysfunctional WAT.

#### Brown Adipose Tissue

Heat production through non-shivering thermogenesis occurs chiefly in the brown adipose tissue (BAT) in different organisms including humans. Therefore, BAT represents a natural target for increasing energy expenditure since thermogenesis relies on energy dissipation to maintain body temperature. This process depends on the specific expression in brown adipocytes of the uncoupling protein UCP1, a mitochondrial inner membrane protein that promotes dissipation of nutrient-derived energy in the form of heat [reviewed in (Cannon and Nedergaard, 2004)]. Therefore, although insulin regulates metabolism in both brown and white adipocytes, the role of both tissues in energy storage and utilization is quite different. Unlike WAT, BAT accumulates lipids not as a store for the excess of energy but as a source of molecules to be oxidized in mitochondria when thermogenesis is activated to produce heat (Guillen et al., 2013). Given the role of BAT as a sink for draining and oxidation of glucose and triglycerides from blood, enhancing BAT development/activity or promoting browning of WAT may contribute to a reduction in body weight and to the improvement of glucose tolerance.

GRK2 appears to play an important role in both BAT function and architecture, as well as in brown adipocyte differentiation (Vila-Bedmar et al., 2012). In keeping with this notion, the decreased age-induced weight gain observed in adult GRK2+/− mice seems to be due, at least in part, to a preserved function of BAT in these animals. Accordingly, GRK2 hemizygous mice display higher energy expenditure and lower respiratory exchange ratio. This correlates with morphology of GRK2+/− BAT that is more consistent with an efficient thermogenic function (Vila-Bedmar et al., 2012). Moreover, decreasing GRK2 during a HFD through tamoxifen-induced genetic depletion prevents fat accumulation and increases the expression of fatty acid oxidation and thermogenic markers within BAT (Vila-Bedmar et al., 2015). Furthermore, BAT explants of these animals showed an enhanced *ex vivo* lipolytic response to the β-adrenergic agonist isoproterenol. Altogether, these data point to an increased capacity for fatty acid metabolism in mice with low GRK2 levels and might explain the absence of lipotoxicity observed in these animals despite the increased free fatty acid availability caused by enhanced WAT lipolysis. They also point toward GRK2 inhibition as a potential tool for the enhancement of brown fat activity that could have additive effects to the function of this kinase in the regulation of insulin signaling.

### Liver

The liver plays a central role in the regulation of metabolic homeostasis. For instance, the synthesis of glucose from non-carbohydrate sources during nutrient deprivation or fasting occurs in the liver through gluconeogenesis. When refeeding allows glucose levels to rise, hepatic gluconeogenesis is inactivated and the liver stores ingested carbohydrates as glycogen. Insulin signals restore the normoglycemia after a meal chiefly by regulating these two processes, gluconeogenesis and glycogen synthesis, in the liver. However, in metabolic disorders, the liver develops IR leading to increased glucose output and decreased glucose clearance leading to sustained hyperglycemia that can bring about pathological consequences (Czech, 2017).

One of the most important complications related to hepatic IR is non-alcoholic fatty liver disease (NAFLD) that expands from simple steatosis to non-alcoholic steatohepatitis (NASH). NASH involves the establishment of inflammation, fibrosis, and cirrhosis in the liver, which can originate end-stage liver failure and eventually evolve into hepatocellular carcinoma (Malaguarnera et al., 2009). One major underlying cause of NASH is the development of hepatic IR, a feature that upregulates the levels of hepatic lipogenic transcription factors and underlies triglyceride accumulation in the liver thus causing hepatic steatosis (Malaguarnera et al., 2009; Smith and Adams, 2011; Cusi, 2012).

Interestingly, chronic insulin stimulation increases GRK2 levels and decreases insulin receptor expression in mouse liver FL83B cells (Shahid and Hussain, 2007). This is in accordance with the absence of an activation of glycogen synthesis following the defective phosphorylation status of IRS1 observed in these cells (Shahid and Hussain, 2007). In keeping with these results, our group has shown that GRK2 levels were increased in the liver of mice fed a HFD (Garcia-Guerra et al., 2010) or a methionine and choline-deficient diet (MCD), as a mouse model of NASH, and also in human patients diagnosed with NASH (Cruces-Sande et al., 2018). Also, Sprague-Dawley rats fed a HFD present increased hepatic plasma membrane-associated GRK2 (Charbonneau et al., 2007). Interestingly, insulin-mediated signaling is maintained in the liver of GRK2 hemizygous mice under different IR-inducing conditions (Garcia-Guerra et al., 2010). Moreover, decreasing GRK2 during a HFD by means of tamoxifen-induced genetic depletion prevents hepatic steatosis and shifts the expression of proinflammatory toward anti-inflammatory markers in the livers of these animals (Vila-Bedmar et al., 2015). These data uncover a role for GRK2 in the regulation of fat accumulation and inflammation in the liver. A very recent study also reveals that mice partially deficient for GRK2 are resistant to the development of NASH independently of obesity and IR (Cruces-Sande et al., 2018). These animals were diagnosed with simple steatosis and not NASH after an MCD feeding and present lower inflammation, a better handling of ER stress, preserved autophagy, and more active processes of mitochondrial biogenesis and dynamics after the MCD.

## Organ Crosstalk in Cardiovascular and Metabolic Diseases

It is worth noting that cardiovascular and metabolic diseases are often present as co-morbidities and the tissues and organs involved in these pathological situations are highly interconnected. Type-2 diabetes and obesity enhance the chance of developing heart failure independently of other risk factors (Riehle and Abel, 2016) by mechanisms involving altered systemic neurohormonal, metabolic, hormonal, and inflammatory mediators (enhanced catecholamine and angiotensin levels, hyperinsulinemia, hyperglycemia, augmented circulating free fatty acids (FFA), or altered adipokine and cytokine secretion), contributing to altered cardiac metabolism and signaling, and fostering the development of diabetic cardiomyopathy (Woodall et al., 2014; Jia et al., 2016). NAFLD has also been linked to increased risk of cardiovascular disease as a result of aberrant glucose, fatty acid and lipoprotein metabolism, oxidative stress, altered cytokine secretome, and endothelial dysfunction (Bhatia et al., 2012). In addition, emerging evidence points to additional crosstalk mechanisms from the heart to peripheral organs. The injured and remodeling heart secretes a variety of inflammatory cytokines and metabolic and lipid mediators that can in turn impact the functions of the kidney, adipose tissue, or the liver [reviewed in (Baskin et al., 2014; Fujiu et al., 2017)], thus perpetuating a pathological cycle among the heart and key metabolic tissues. The fact that different pathological triggers converge in promoting GRK2 upregulation in many of the tissues involved in such crosstalk reinforces the notion that simultaneous GRK2 inhibition in all the tissues implicated in a given pathology might have a synergic beneficial effect.

## Strategies to Target GRK2

Genetic inhibition has been, so far, the most successful strategy to achieve a downregulation of GRK2 protein/function as a tool to demonstrate the participation of this kinase in physiopathological processes and as a proof of concept of its potential use as a therapeutic target. A mounting amount of evidence describes the effects of GRK2 downregulation either in mouse models deficient for GRK2, tissue-specific and Cre-based inducible depletion, siRNA technology both in mice and in cultured cells, and also adenoviral and lentiviral transfer of GRK2-specific silencing or overexpression constructs. This has served to establish GRK2 as a target to tackle with important diseases, as recently reviewed elsewhere for cardiac (Cannavo et al., 2018), metabolic (Mayor et al., 2018), vascular (Schumacher and Koch, 2017), renal (Rudomanova and Blaxall, 2017b), and tumoral (Nogues et al., 2017) pathological contexts.

The genetic approaches to downregulate GRK2 levels, although not translatable to the clinical practice, can thus provide very useful information about the feasibility and potential drawbacks of GRK2 as a therapeutic target. In this regard, one particularly interesting model is the use of global hemizygous GRK2 mice, which would *bona fide* mimic the likely partial effects of pharmacological systemic inhibition of GRK2 and show the integrated effects of GRK2 inhibition in different tissues and cell types (Avendano et al., 2014; Lucas et al., 2014). On the other hand, inducible global GRK2 deletion models can help to determine whether systemic GRK2 downregulation can not only prevent but also revert certain diseases such as reported in the context of insulin resistance, overweight, and glucose (Vila-Bedmar et al., 2015). These approaches can also inform of potential disadvantages or possible side effects of GRK2 inhibition and differentiate between effects due to inhibition of kinase activity alone (by using kinase activity inhibitors as detailed below) or also to scaffold functions of this protein (only altered when decreasing protein levels). For instance, a recent publication suggests that a global GRK2 downregulation by using a silencing construct under the control of the U6 mouse polymerase III promoter may have detrimental outcomes in kidney function and development (Tutunea-Fatan et al., 2018), thus suggesting that therapeutic strategies that target GRK2 activity, not expression, could be safer in particular for the treatment of hypertension (Tutunea-Fatan et al., 2015).

More specific targeting strategies such as those reducing GRK2 expression in particular cell types can also provide useful information about possible cell type-specific drawbacks of chronic GRK2 inhibition. For instance, a reduction of GRK2 in myeloid cells increases the risk of septic shock in mouse models (Patial et al., 2011), although myeloid-specific GRK2 does not alter immune cell infiltration to the primary site of infection or bacterial clearance and does not significantly affect mortality in a cecal ligation puncture model of polymicrobial sepsis (Parvataneni et al., 2011). In microglia, GRK2-targeting can transform acute inflammatory pain into chronic hyperalgesia (Eijkelkamp et al., 2010), and decreased GRK2 levels in endothelial cells can cause reduced maturation of vessels (Rivas et al., 2013) and defects in vascular function and structure (Ciccarelli et al., 2013). In sum, although a reduction of GRK2 protein and/or activity is as a clear target for intervention in cardiovascular and metabolic pathologies, care should be taken when exploring the consequences of downregulating GRK2 in other diseases involving renal, myeloid, or endothelial cells.

In addition to genetic approaches, a variety of other potential strategies to inhibit GRK2 functions are being developed based on the structural features and mechanisms of regulation of this kinase and are described in the following sections.

### Small Molecule Inhibitors of GRK2

The search for a potent and selective small molecule inhibitor of GRK2 has turned out to be a difficult quest. The first described compounds able to bind and negatively impact GRK2 activity were polyanionic and polycationic compounds such as heparin (Benovic et al., 1989). They were unable to cross the plasma membrane, but capable of inhibiting *in vitro* GRK2-dependent rhodopsin phosphorylation with IC50 values below μM.

It was not until the crystal structure of human GRK2 in complex with balanol, a fungal metabolite synthetized by *Verticillium balanoides,* was solved (Tesmer et al., 2010) that the mechanism of action of ATP-competitive inhibition of GRK2 was better elucidated and used in the search of compounds with increased selectivity for this kinase relative to other protein kinases and GRK family members [reviewed in (Guccione et al., 2016)]. The relative efficacy of balanol, as tested in *in vitro* kinase assays using tubulin and rhodopsin as substrates, showed certain selectively toward GRK2 and GRK3 (with IC50 in the low nM range for both isoforms) as compared with GRK5 and GRK7 with IC50 in the high nM range, similar to those reported for PKA (Tesmer et al., 2010). Balanol binds to a semi-closed inactive conformation of GRK2 (as compared with PKA) that could be exploited pharmacologically. Since balanol adopts different conformations when binding to GRK2 and to PKA, selectivity for GRK2 could be improved by designing molecules that adapt to the particular conformation of the catalytic site adopted by GRK2 when bound to this compound that could be different for different GRK isoforms.

The Takeda Pharmaceutical Company also identified several heterocyclic compounds that bound and inhibited GRK2, and they were subsequently co-crystallized with GRK2-Gβγ (Thal et al., 2011). Similar to balanol, these compounds (called CMPD101 and CMPD103A) stabilized GRK2 in a slightly closed non-catalytic conformation with a degree of closure that correlated with potency for each species. Their IC50 values in a rhodopsin phosphorylation assay were of 290 nM and 54 nM, respectively (Thal et al., 2011), although an IC50 value of 35 nM had been previously reported in the corresponding patent. CMPD103A and CMPD101 are remarkably more selective among GRK subfamilies than balanol since they inhibit GRK2 and GRK3 (“GRK2 subfamily”) with IC50s in the nM rage for both isoforms (that share 92% identity in their kinase domain sequences), but do not have an effect on GRK1 or GRK5 isoforms when used in concentrations up to 125 μM (Thal et al., 2011). However, they inhibited PKA with an IC50 of 2 μM. Possibly because of bioavailability problems, these compounds have not yet reached clinical trials even when they show desensitization-blocking activity and good potency in cellular assays [see (Rainbow et al., 2018) as an example]. Through a high throughput screening identification strategy using Ulight TopoIIα as an artificial substrate and subsequent SAR-guided development, the same company has more recently developed a novel class of GRK2 inhibitors with selectivity toward isoforms GRK1, 5, 6, and 7, but showing equipotent inhibition of GRK3 (Okawa et al., 2017). Crystal structures using human GRK2 show a ligand-binding pose and interactions similar to those previously observed (Thal et al., 2011). The best performing compound was named 115 h, has an IC50 for GRK2 of 18 nM, and blocks desensitization of the β-adrenergic receptor pathway in HEK293 cells, although these effects were shown only at 100 μM concentrations. The pharmacokinetic profile after oral administration was however not enough to show *in vivo* activity.

An emerging family of small molecules being developed as GRK2 inhibitors are based on the FDA-approved selective serotonin reuptake inhibitor (SSRI) paroxetine, which was first found to target GRK2 in an aptamer displacement assay (Thal et al., 2012). Paroxetine stabilizes a unique conformation of GRK2 that misaligns the small and large lobes of this kinase and thus represents a unique scaffold for the design of more selective GRK2 inhibitors (Thal et al., 2012; Homan and Tesmer, 2015). Even when the IC50 of paroxetine is in the μM range using the rhodopsin (≈20 μM) or tubulin (≈2 μM) *in vitro* phosphorylation assays, the selectivity toward other GRK isoforms was a great improvement. Paroxetine was 50 to 60 times more potent toward GRK2 than toward GRK1 and GRK5 and performed well in living cells with a 10- or 40-fold selectivity over PKA and PKC, respectively (Thal et al., 2012). Paroxetine was also shown to inhibit β-adrenergic receptor desensitization by blocking GRK2-mediated receptor phosphorylation and β-arrestin recruitment with an IC50 of ≈6 μM (Guo et al., 2017). *In vivo*, paroxetine has been described to improve LV function and structure and to reverse or even ameliorate several features related to cardiac dysfunction in a mouse model of myocardial infarct when compared with fluoxetine, a structural analog unable to inhibit GRK2 but with SSRI capacity (Schumacher et al., 2015). Also, in a rat model of limb ischemia-reperfusion (I/R) injury, decreased GRK2 expression levels were found in ipsilateral neurons of the superior cervical ganglion. However, in mice treated with paroxetine, GRK2 expression was preserved after I/R, thus linking GRK2 binding/inhibition with the stabilization of the protein (Tang et al., 2015). Moreover, in another rat model of collagen-induced arthritis, oral (gavage) administration of paroxetine showed *in vivo* effects by protecting the joints from inflammation and destruction by impairing T cell infiltration and activation (Wang et al., 2017b). Cytokine and chemokine levels in serum and synovial tissues were also reduced, as were the populations of CD4+ and CD8+ effector T cells with increased differentiation of Treg cells and an induction of immune tolerance. Altogether, these results show that this drug can have pharmacological effects in murine models even when using enteral administration.

When a library of known kinase inhibitors from the Structural Genomics Consortium at Oxford University was screened, several compounds targeting GRK2 with structures resembling paroxetine were found (Homan and Tesmer, 2015). The most active compound showed an IC50 below μM in the tubulin phosphorylation assay and was 100–1,000 times more potent toward GRK2 over GRK1 and GRK5 (Homan and Tesmer, 2015). A highly potent and selective GRK2 inhibitor called 14as has been more recently identified from a set of paroxetine-derivative compounds (Waldschmidt et al., 2016). 14as has an IC50 for GRK2 of 30 nM, shows more than 230-fold selectivity over GRK1, GRK5, PKA, and ROCK1, and performs two orders of magnitude better than paroxetine in cardiomyocyte contractility assays, although no data relative to the possible inhibition of the GRK3 isoform are shown or discussed. Co-crystal structures of three of the synthesized paroxetine derivatives revealed the establishment of hydrogen bonds that make GRK2 adopt a more open conformation relative to that achieved with other inhibitors, which probably underlies the high selectivity of these compounds. More interestingly, pharmacokinetic data indicate that its plasma concentration in mice after a single intraperitoneal administration is above its IC50 for over 7 h.

The molecule GSK180736A, a compound structurally similar to paroxetine that had been developed as a ROCK1 inhibitor, was shown to co-crystallize in the active site of GRK2 and is a potent and selective inhibitor of GRK2 with an IC50 of 770 nM and more than 100-fold selectivity over other GRK isoforms. This molecule was used to develop a library of hybrid inhibitors containing some features from the Takeda compounds, namely occupation of the hydrophobic binding site in the kinase domain of GRK2, together with others from the GSK180736A molecule (Waldschmidt et al., 2016). From this library, inhibitors that are highly selective for GRK2 as well as potent for both GRK2 and GRK5 emerged. In particular, the compound called 12n (CCG-224406) showed an IC50 of 130 nM for GRK2 and, remarkably, more than 700-fold selectivity over GRK1 and GRK5, although no comparison using GRK3 is shown in this study. Emerging from other two classes of GRK2-selective inhibitors, namely GSK180736A and paroxetine, a molecular design initiative led to the creation of a set of new hybrid compounds in which the benzodioxole ring of paroxetine was exchanged for an indazole (indazole-paroxetine hybrids) (Bouley et al., 2017). Crystal forms from these hybrid molecules showed that they not only form stronger interactions with the hinge of GRK2 but also stabilize a distinct conformation, compared with paroxetine analogs, of its kinase domain. Therefore, the binding modes of these two families of compounds to the active site of GRK2 are similar, but they use two different hinge-binding moieties: indazole and benzodioxole. Among them, the CCG224061 compound presents a 20-fold increase in potency for GRK2 (IC50 of 66 nM) over paroxetine. However, it also shows increased activity against GRK1 and 5, PKA, and ROCK1 relative to the latter compound, and the results relative to GRK3 inhibition are not shown. So, the indazole-paroxetine analogs were more potent than benzodioxole derivatives, however at the cost of poorer selectivity, and possibly with a particular pharmacokinetic profile that may include renal clearance.

A very recent study has utilized some of the most active among the formerly presented GRK2 inhibitors to test GRK2-mediated desensitization of different arterial vasoconstrictor stimuli using *ex vivo* approximations (Rainbow et al., 2018). Paroxetine, Takeda’s CMPD101, and two amide derivatives of GSK180736A (Waldschmidt et al., 2016) termed CCG215022 and CCG224063 were used. Each of these compounds attenuated desensitization toward angiotensin II- or UTP-mediated contractile responses in myograph assays using rat arterial rings, as well as in P2Y_2_ and H_1_ histamine-mediated PLCβ signaling assays, in MSMC and ULTR cells with an IC50 in the low μM range (CCG224063 in the low nM). Altogether these and other functional and structural data are unveiling interesting molecular insights into the binding modes, kinase selectivity and pharmacological features of these families of GRK inhibitors.

### Aptamers

Since the first RNA-based aptameric molecule that acts as an *in vitro* inhibitor of GRK2 activity was described (Mayer et al., 2008), several efforts to improve the potency and selectivity of this class of compounds have been pursued. This C13 molecule can stabilize a unique inactive conformation of GRK2 through different interactions within and outside the kinase domain and reorganizes certain regions of the kinase to establish interactions with its polyanionic phosphodiester backbone (Mayer et al., 2008). C13 achieves inhibition of rhodopsin phosphorylation by GRK2 at low nM concentrations with a 20-fold selectivity over GRK5, and no appreciable effects detected in a panel of 14 other kinases (Mayer et al., 2008). However, possible effects on a cellular setting have not yet been reported, and this is of particular concern since these types of molecules are not bioavailable when administered orally, may not be stable inside the organism due to endoribonuclease-driven cleavage, and do not readily cross cell membranes. So, medicinal chemistry efforts should be implemented in order to overcome these difficulties. They have however been successfully used as a method of selection to screen for better drug-like inhibitory molecules by aptamer displacement assays as described above.

### Peptides

Other strategy to inhibit GRK2 activity is based on using peptide sequences to interfere with the interface between the kinase and its substrates or activators. Peptides corresponding to the β2-adrenergic receptor sequence reported to interact with GRK2 have been identified and subsequently modified (Benovic et al., 1990) to reduce kinase activity with an IC50 in the low μM range but showing varying degrees of selectivity among GRK isoforms. Interestingly, these peptides do not seem to interact broadly with the kinase domain of GRK2, but rather mimic the first intracellular loop of the receptor and establish only one contact point with the kinase. One of them shows activity in cells, impairs β2-adrenergic receptor desensitization, and enhances receptor signaling in the human A431 cell line. Other peptides derived from residues of the substrate-kinase interaction domain in GRK2 and GRK3 have been tested in several animal models of type 2 diabetes. Acylated glycine derivatives of these short peptides, such as KRX-683_107_ and KRX-683_124_, can reduce plasma glucose concentrations possibly through GRK2-mediated inhibition of insulin signaling and display a systemic antidiabetic effect (Anis et al., 2004; Cipolletta et al., 2009). Another group of peptides was identified from a library of cyclic peptides designed using the GRK2 crystal structure, in particular the HJ loop inside the catalytic fragment of this kinase (Winstel et al., 2005). Peptides 9b and 10d showed sub μM values of IC50 when tested in an *in vitro* rhodopsin phosphorylation assay with poor inhibition of GRK5 and also presented activity in cells increasing basal and isoproterenol-stimulated cAMP production in HEK-293 cells overexpressing β2-adrenoreceptors.

Rational design of inhibitory peptides taking advantage of allosteric intramolecular interactions in GRKs has also been studied. For instance, N-terminal interactions as well as those emerging from an interface able to stabilize the closed active conformation of GRK2 (where receptor binding is proposed to activate GRK2) have been explored (Huang et al., 2009). A peptide encompassing the first 14 residues of GRK2 decreases receptor phosphorylation with an IC50 of 50 μM and at the same time enhances binding to phospholipids in what appears to be a membrane-specific effect since it has no impact on the kinase activity toward the soluble substrate tubulin (Pao et al., 2009).

### Targeting the Gβγ-GRK2 Interface

The overexpression of a C-terminal fragment of GRK2 called GRK2ct or βARKct has been used as a strategy for GRK2 inhibition, since this peptide should inhibit endogenous GRK2 binding to Gβγ subunits and thus subsequent activation and translocation to the plasma membrane. Importantly, transgenic mouse models expressing βARKct constructs in the heart or adenoviral-mediated delivery of this construct in mice and pig models has shown to be cardioprotective in both acute and chronic models of HF. Since these aspects have been recently reviewed (Rudomanova and Blaxall, 2017b; Campbell and Smrcka, 2018; Cannavo et al., 2018), we will only discuss herein potential future developments of this strategy and also some caveats regarding its functional impact.

Since viral-based gene delivery remains a daunting therapeutic methodology, efforts have been made to identify small molecule Gβγ interactors as a more viable alternative. Consequently, a virtual screening was performed on a National Cancer Institute chemical library to identify small molecules capable of binding Gβγ subunits, using as an assay the ability to displace Gβγ binding to SIGK, a peptide that has been co-crystallized with the dimer, and thus identifying a surface critical for Gβγ interaction with effectors (Campbell and Smrcka, 2018). Among those, one termed M119 (cyclohexanecarboxylic acid [2-(4,5,6-trihydroxy-3-oxo-3H-xanthen-9-yl)-(9Cl)]) demonstrated high apparent affinity for Gβγ dimers and inhibited Gβγ-SIGK binding *in vitro* (Bonacci et al., 2006). The compound M119 and its highly homologous and more stable analog gallein have been successfully used ever since both in cultured cells and *in vivo*. They are able to impair Gβγ-dependent GRK2 recruitment to the plasma membrane in response to GPCR in different cell types. Moreover, they can hamper HF progression and improve cardiac function in different murine models where they halt fibrosis, hypertrophic gene expression, and inflammation and also reduce myocardial infarct size [reviewed in (Rudomanova and Blaxall, 2017a)]. Also, in this context, a group has recently reported the development of nanobodies that able to specifically inhibit Gβγ-dependent signaling (Gulati et al., 2018).

It is worth noting that either GRK2ct or these new types of compounds would not only affect Gβγ-GRK2 activation but also block other Gβγ effectors downstream GPCR activation, which may cause off-target effects that need to be considered when evaluating their therapeutic roles. Although the notion of selective delivery of Gβγ inhibitors to specific target tissues using viral-guided approaches is attractive, the possible clinical application of this kind of strategies faces many regulatory and technical issues that need to be solved.

### Emerging Strategies to Target GRK2 Functionality

The in-depth characterization of GRK2 modulatory mechanisms and the identification in recent years of a variety of new cellular partners of this kinase help envisage novel targeting strategies based on processes that able to modulate GRK2 activity/expression or to control specific functions of GRK2.

One type of approach is based on the modulation of GRK2 activation and translocation to the plasma membrane. Similar to the already discussed inhibitory effect caused by disruption of the interaction between GRK2 and Gβγ subunits, the GRK2^K567E/R578E^ mutation, which eliminates anionic phospholipid binding, also ablates receptor phosphorylation in cells (Carman et al., 2000). In fact, efficient GRK2-mediated phosphorylation of activated GPCRs is dependent not only on its recruitment to the membrane by Gβγ subunits but also on the presence of phosphatidylinositol 4′,5′-bisphosphate (PIP2), a highly negatively charged lipid that not only helps recruit GRK2 but also orients the GRK2-Gβγ complex so that it is better able to phosphorylate activated GPCRs (Yang et al., 2016). This result is in contrast to what occurs with the GRK5 isoform, which adopts similar orientations on lipid bilayers whether or not they contain PIP2 (Yang et al., 2013). Therefore, isoform-selective inhibitors could be designed to target the GRK2-phospholipid interaction and thus impair or redirect kinase activity toward defined substrates (e.g., soluble vs membrane bound). However, other studies have described the ability of phosphatidylserine (PS) to enhance GRK2-dependent phosphorylation of purified and reconstituted human m2 mAChR twofold to threefold while PIP2 strongly inhibited this reaction (DebBurman et al., 1995). A possible explanation for this apparent discrepancy may be that neither Gβγ nor phospholipid interactions appear to play a major role in GPCR phosphorylation by GRK2 *in vitro*, possibly because high concentrations of the kinase and/or receptor can be used to drive the reaction by mass action and thus lead to apparently contradictory results. Since this type of GRK2-plasma membrane *in vivo* interactions may not be accurately mimicked by *in vitro* systems, cell-based assays should be utilized in order to develop inhibitors based on the ability to interfere with lipid-dependent GRK2 activity. In any case, the fact that GRK2 can be considered a lipid-dependent kinase that may be both upregulated and downregulated by phospholipids is an interesting feature that should not be overlooked when suggesting new strategies for the regulation of its activity.

Other strategies may rely on the interaction of GRK2 with inhibitory proteins. RKIP, a multifaceted kinase modulator belonging to the conserved family of phosphatidylethanolamine-binding proteins (PEBPs), is a small 21 kDa protein that able to regulate different signaling cascades and physiological processes. Phosphorylation of RKIP S153 by PKC appears to reorganize RKIP domains from a structure that binds and inhibits Raf-1 into a conformation that associates and blocks GRK2 by binding to its N-terminus domain [reviewed in (Skinner and Rosner, 2014)]. The inhibitory mechanism possibly requires RKIP dimer formation (Deiss et al., 2012). In fact, overexpression of phosphomimetic (RKIP^SK153/7EE^) or dimeric (RKIP^Δ143–146^) RKIP peptides impairs phosphorylation of βARs by GRK2 in HEK293 cells as detected using antiphosphoserine antibodies and also reduces GRK2-mediated rhodopsin phosphorylation *in vitro* (Deiss et al., 2012). This study also shows that, at least in murine hearts, RKIP exists mostly in the S153-phosphorylated form and is thus predominantly bound to GRK2. In keeping with these data, interfering with RKIP by using specific antibodies, antisense, or RNAi plasmids enhances β-adrenergic signaling and its related contractile activity in cardiomyocytes (Lorenz et al., 2003). Moreover, a modest overexpression of this protein in a transgenic mouse model produces an increase in cardiac contractility achieved by the simultaneous activation of the β_1_AR and β_2_AR subtypes of adrenergic receptors that is thus well-tolerated and persistent (Schmid et al., 2015). These results open the possibility to use phosphorylated/dimeric RKIP to interfere with GRK2 functions in manners that could be alternative or additive not only to the use of pharmacological GRK2 blockade but also to study new modes to achieve GRK2 functional downregulation. However, given the multifaceted role of this peptide in the control of other important kinases and pathways such as Raf, MEK, ERK, NFκB, GSK3β, among others, the selectivity of such strategies could be compromised and should be carefully examined. Finally, since other proteins such as caveolin1, actinin, or clathrin have been reported to bind to GRK2 and help maintain pools of inactive kinase at defined subcellular locations (Penela et al., 2010a), it is tempting to suggest that peptides derived from these regulatory partners (or small molecules mimicking those) may modify the catalytic activity of GRK2 and/or its subcellular distribution, thus selectivity affecting certain GRK2 substrates.

On the other hand, post-translational mechanisms activating/inhibiting GRK2 or switching its partner repertoire could potentially be used to interfere with GRK2-dependent cellular actions. As previously mentioned, GRK2 activity is regulated in opposing ways by different covalent modifications, such as those entailing tyrosine phosphorylation of GRK2 within the N-terminus and the RH domain, Ser670 phosphorylation, or cysteine-nitrosylation in the catalytic domain. In particular, the S-nitrosylation of GRK2 limits the ability of this kinase to phosphorylate and desensitize the β2AR [recently reviewed in (Cannavo and Koch, 2018)]. Accordingly, mice carrying a point mutation that substitutes the Cys340 in GRK2 for a serine (GRK2^C340S^) are less resistant to cardiac ischemic injury and lose responsiveness of their myocardium to increased or decreased NO (Huang et al., 2013). Moreover, a water-soluble N-nitrosamine able to nitrosylate GRK2 without generating NO has been shown to impair isoproterenol-mediated β2AR-desensitization *via* GRK2 inhibition (Makita et al., 2013), thus providing a proof of concept that S-nitrosylation of GRK2 can be used as a therapeutic strategy.

Modulation of upstream kinases may also affect GRK2 functionality in specific contexts. In particular, our group has reported that GRK2-mediated HDAC6 phosphorylation requires the previous phosphorylation of GRK2 in S670 (see previous sections), which apparently provokes a switch in GRK2 substrate usage (Lafarga et al., 2012). GRK2^S670A^ showed a noticeably reduced ability to phosphorylate HDAC6 as compared with WT GRK2 but did not show any defects in phosphorylating other GRK2 canonical substrates. This discovery opens the possibility for redirecting GRK2 activity toward defined substrate subsets. For instance, the use of compounds or cellular strategies that impair GRK2 phosphorylation in S670, such as ERK or CDK2 inhibitors, would either hamper or completely impair phosphorylation of HDAC6, and thus, GRK2-dependent effects downstream this deacetylase such as growth factor signaling, proliferation, and anchorage-independent growth of breast cancer cells (Nogues et al., 2016). Although not specifically targeted to GRK2, such ERK or CDK2 inhibitors would indirectly rewire the GRK2 substrate repertoire by affecting its Ser670 phosphorylation status. In this context, it would be of interest to identify small molecules that differentially inhibit the activity of unphosphorylated and Ser670-phosphorylated GRK2 toward distinct cellular substrates. Other possible approach to impair GRK2 activity toward given substrates might be the use of peptides (or small molecules) targeting the specific domains of GRK2 (or of its partner) involved in their recognition. Also, the substrate preference of GRK2 would depend on its particular subcellular localization in a given cellular setting. For instance, GRK2 can be localized to the mitochondria by way of its interaction with Hsp90 proteins triggered by S670 phosphorylation (Chen et al., 2013), and an important pool of microsomal-bound GRK2 has been described in mouse liver and neuronal tissues as well as in cultured cells (Murga et al., 1996). The consequences of the existence of different subcellular populations of GRK2 in terms of accessibility to defined substrates and the possibility that they may cause different final downstream effects are only beginning to be studied and should be fully elucidated.

A better knowledge of the stimuli and mechanisms controlling GRK2 expression may help to develop approaches to directly decrease GRK2 expression. As discussed in previous sections, diverse stimuli (catecholamines, angiotensin, high-fat diet) appear to converge in promoting enhanced GRK2 expression in different tissues and cell types in the context of given diverse cardiovascular and metabolic diseases. Thus, it is tempting to suggest that altering such stimuli would help to prevent pathological GRK2 accumulation. In fact, beta-blockers as well as exercise have been described to reduce myocardial and vascular GRK2 levels [reviewed in (Hullmann et al., 2016; Cannavo et al., 2018; Mayor et al., 2018)]. On the other hand, the mostly unexplored field of GRK2 expression modulation by miRNAs may also identify tools for decreasing kinase levels in a tissue-specific way. Finally, the well-established degradation of GRK2 *via* the ubiquitin-proteasome pathway may also allow for strategies aimed at increasing GRK2 downregulation in particular cellular settings such as proteasome activators or the use of emerging techniques to target specific proteins for degradation, such as the use of the so-called Proteolysis Targeting chimeric molecules (PROTACs) (Gu et al., 2018).

## Conclusions

The GRK2 signaling hub is important in signaling pathways and processes related to very relevant cardiovascular pathological conditions (heart failure, cardiac hypertrophy, hypertension) and to diseases related to altered metabolic homeostasis (obesity metabolic syndrome, type 2 diabetes, NAFLD). These data along with the increased GRK2 expression reported in preclinical models of these pathologies and in samples from patients put forward this kinase as a promising therapeutic target. Moreover, the fact that these pathological conditions are frequently interconnected suggests that inhibiting GRK2 may have common beneficial effects when such situations concur as co-morbidities. In fact, genetic approaches have established the importance of GRK2 as a potential therapeutic target in some of these pathological processes. Strategies aimed to target GRK2 are beginning to yield some fruits, and several small molecules, peptides, and inhibitory constructs have been developed that effectively inhibit GRK2 activity and have been shown to display effects in cells and even in animal models. However, there are many challenges that need to be addressed. In addition to attaining specificity toward other GRKs and other kinases and off-targets, GRK2 inhibitors with acceptable *in vivo* potency and pharmacokinetic profiles are still awaited. On the other hand, the potential drawbacks of pathological effects of chronic GRK2 inhibition in defined cell types and tissues need to be carefully considered in order to identify therapeutic windows. In addition, the identification of novel GRK2 substrates and partners and the existence of different subcellular pools open the possibility of specifically targeting the interaction of GRK2 with specific subsets of signaling proteins. A better knowledge of the dynamic structural events leading to GRK2 activation, of the interfaces involved in its interaction with given partners, and of the molecular mechanisms involved in the modulation of GRK2 expression, activity, and localization would help to develop or improve novel strategies for GRK2 inhibition. This would be very important not only for advancing in the path for therapeutic application but as advanced research tools to dissect the precise contribution of GRK2 to physiological and pathological processes.

## Author Contributions

All authors listed have made a substantial, direct and intellectualcontribution to the work, and approved it for publication.

### Conflict of Interest Statement

The authors declare that the research was conducted in the absence of any commercial or financial relationships that could be construed as a potential conflict of interest.
